# Proteome difference among the salivary proteins adsorbed onto metallic orthodontic brackets and hydroxyapatite discs

**DOI:** 10.1371/journal.pone.0254909

**Published:** 2021-07-28

**Authors:** Walter Luiz Siqueira, Maria Pia Canales, Karla Tonelli Bicalho Crosara, Lina Maria Marin, Yizhi Xiao

**Affiliations:** 1 College of Dentistry, University of Saskatchewan, Saskatoon, SK, Canada; 2 Schulich Dentistry & Medicine, The University of Western Ontario, London, ON, Canada; Virginia Commonwealth University, UNITED STATES

## Abstract

The aim of this study was to investigate the atomic composition and the proteome of the salivary proteins adsorbed on the surface of orthodontic metallic bracket. For this, the atomic composition of orthodontic metallic brackets was analyzed with X-ray Photoelectron Spectroscopy (XPS). The acquired bracket pellicle was characterized after brackets were immersed in human whole saliva supernatant for 2 hours at 37°C. Hydroxyapatite (HA) discs were used as a control. Acquired pellicle was harvested from the HA discs (n = 12) and from the metallic brackets (n = 12). Proteomics based on mass spectrometry technology was used for salivary protein identification and characterization. Results showed that most of the proteins adsorbed on the surface of orthodontic metallic brackets and on the HA discs were identified specifically to each group, indicating a small overlapping between the salivary proteins on each study group. A total of 311 proteins present on the HA discs were unique to this group while 253 proteins were unique to metallic brackets, and only 45 proteins were common to the two groups. Even though most proteins were unique to each study group, proteins related to antimicrobial activity, lubrication, and remineralization were present in both groups. These findings demonstrate that the salivary proteins adsorbed on the bracket surface are dependent on the material molecular composition.

## Introduction

A constant concern in the orthodontic clinic is the high prevalence of active caries lesions in patients undergoing treatment. In fact, it has been noted that 30 to 70% of patients experience active caries lesions during orthodontic treatment [1, 2]. Although dental caries is a multifactorial disease, the characteristics of the biofilm are decisive for the development of the condition. Therefore, understanding the factors that dictate the bacterial colonization on orthodontic devices is crucial in the search for new preventive strategies against the progression of dental caries during orthodontic treatment.

Several studies have looked at the factors that guide bacterial colonization on tooth surface [3–13]. Among such factors, the composition of the acquired enamel pellicle (AEP) [7–11], a thin protein layer formed on the tooth surface [14], is extremely important. The AEP plays a key role in the maintenance of oral health by regulating processes such as lubrication of the oral cavity, and tooth demineralization and remineralization [15]. Furthermore, the AEP also acts as the foundation to which bacteria selectively adhere to the enamel surface. Therefore, the composition of the AEP is fundamental in determining the cariogenicity of the formed biofilm.

Previous studies indicated that the level of bacterial growth on the surface of the orthodontic brackets varies according to the material of the bracket -[16–18]. In fact, composite brackets have been reported to attract bacterial colonization at a higher rate than other bracket types, such as metallic brackets [19]. Since orthodontic treatment lasts for an average of two years, the composition of the protein pellicle on the bracket surface, and the consequent biofilm formation, will have an important impact on the abundance of specific oral microorganisms, and, consequently, on the patient’s risk for dental caries.

Salivary pellicles are not limited to the surface of the tooth, they are also present on the oral mucosa [20], on prosthetic restorations [21], and on dental appliances [22]. Curiously, very little is known about the composition of the salivary protein pellicle formed on orthodontic brackets. Through proteomic analysis, the salivary components of the AEP were identified [23]. Similar approach can be used to characterize the protein pellicle specific to orthodontic brackets.

The mechanisms by which proteins adsorb to surfaces is heavily determined by physical-chemical properties of the surface [24]. It is established that minimum changes in the chemical properties of solid surfaces, such as hydroxyapatite (HA), have influence on the adsorption behavior of salivary proteins to the surfaces [19]. For example, when HA discs are treated with sodium fluoride solution (1, 2 or 5%) for 2 hours, the abundance of important salivary proteins related to demineralization and remineralization of the enamel, such as statherin and histatin 1, decreases with increasing concentration of sodium fluoride, suggesting that these proteins are repulsed when HA surface is coated with sodium fluoride [19].

The purposes of this study were to investigate the proteome composition of the acquired protein pellicle formed on the surface of metallic orthodontic brackets, and to compare the bracket pellicle against the pellicle formed on enamel-like surfaces (HA).

## Materials and methods

### X-ray Photoelectron Spectroscopy (XPS)

**Fig 1:**
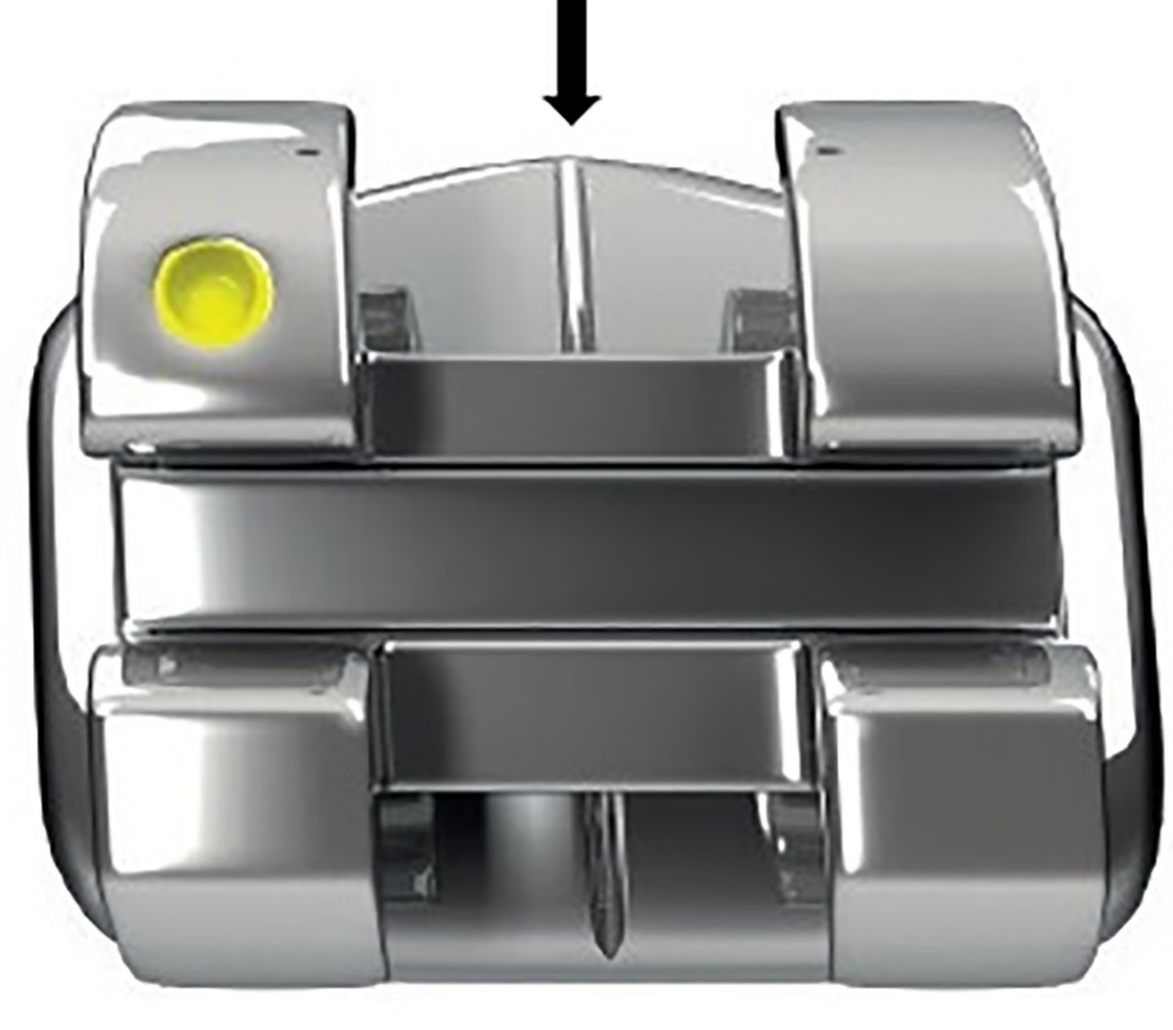
Area of the bracket where the XPS analysis was performed (arrow).

An X-ray Photoelectron Spectrometer (Kratos Axis Ultra Spectrometer) was used to atomically analyze the composition of orthodontic metallic brackets (1.1/2.1 brackets, American Orthodontics, Sheboygan, WI, USA) and HA discs (5 mm dia x 1.5–1.8 mm thick Himed inc., Old Bethpage, NY, USA). Three brackets and three discs were cleaned by sonication, in 800 μL distilled water, for 5 min, and incubated for 2 h at 37°C in 800 μL MiliQ water. Subsequently, XPS atomic analysis was performed on the brackets and HA discs. The XPS equipment was calibrated with the same parameters for all measurements [25]. The instrument work function was calibrated to give a binding energy (BE) of 83.96 eV for the Au 4f7/2 line for metallic gold and the spectrometer dispersion was adjusted to give a BE of 932.62 eV for the Cu 2p3/2 line of metallic copper. All brackets were analyzed in the same area (Fig 1).

### Whole saliva collection

Ethics approval was obtained from the Ethics Committee on Human Research at The University of Western Ontario (protocol number 16181E). All participants signed an informed consent. Clinical evaluation was performed to assure that all participants were free of tooth decay, periodontal disease or any other condition that could affect the salivary composition. A total of 10 mL of whole saliva from three healthy subjects (2 females and 1 male), aged from 30–40 years old, was collected in the morning, between 9:00 AM and 10:00 AM, under masticatory stimulation using parafilm (25cm^2^). Subjects refrained from eating or drinking two hours before saliva collection. After collection, all samples were centrifuged at 14,000 x g for 20 min at 4°C. Following the centrifugation, whole saliva supernatant (WSS) was pooled for the next experiment. Protein concentration was measured by the bicinchoninic acid assay (Pierce Chemical, Rockford, IL, USA).

### Incubation of metallic brackets with human saliva

After sonication in distilled water for 5 min, four metallic brackets (1.1/2.1 brackets, American Orthodontics, Sheboygan, WI, USA) and four HA discs (5 mm dia x 1.5–1.8 mm thick Himed inc., Old Bethpage, NY, USA) were incubated in 800 μL of distilled water for 2 h at 37˚C with gentle agitation (in 24 well culture plate). Subsequently, the same brackets and HA discs were incubated for 2 h at 37˚C in pooled WSS containing the equivalent of 800 μg of total protein (200 μg of protein/bracket or disc). Immediately after incubation, the HA discs and brackets were washed using distilled water to remove any weak binding salivary protein. Experiments were done in triplicate.

### Harvesting of *in vitro* formed pellicles

Pellicle formed on the surface of brackets and HA discs was harvested by sonication in 300 μL of solution containing 80% acetonitrile, 19.9% water and 0.1% trifluoroacetic acid (TFA) for 5 min. This procedure was repeated three times. The eluted pellicle proteins from the three replicates from the same group (metallic brackets or HA discs) were pooled, respectively, and concentrated by a rotary evaporator. Samples were re-suspended in distilled water to 900 μL, and the total protein concentration was assessed by the micro bicinchoninic acid assay (Pierce Chemical, Rockford, IL, USA).

### In–solution digestion

Aliquots of 15 μg of harvested pellicle protein from each group were prepared. The dried samples were re-suspended in 50 μL of 4 M urea, 10 mM DTT and 50 mM ammonium bicarbonate at pH 7.8 and incubated for 1 hour at room temperature. Afterwards, 150 μL of 50 mM ammonium bicarbonate was added to all samples, followed by 3% (w/w) trypsin (Promega, Madison, WI, USA). Thereafter, samples were incubated for 16 h at 37°C. Finally, samples were dried in a rotary evaporator, de-salted by C-18 ZipTip® Pipette Tips (Millipore, Billerica, MA, USA), and subjected to mass spectrometry.

### LC-ESI-MS/MS analysis

After trypsinization, samples were re-suspended in 97.5% distilled water/2.4% ACN/0.1% formic acid and then subjected to reversed-phase nano-liquid chromatography electrospray ionization tandem mass spectrometry (nLC-ESI-MS/MS), using a LTQ-Velos (Thermo Scientific, San Jose, CA, USA) mass spectrometer. Liquid chromatography separation was achieved using a C18 column of capillary fused silica (column length 10 mm, column id 75 m, 3 m spherical beads, and 100 A pores size), linked to the mass spectrometer through electrospray ionization. Peptides were eluted from the nanoflow RP-HPLC over a 65-min period, with linear gradient ranging from 5 to 55% of solvent B (97.5% ACN, 0.1% formic acid), at a flow rate of 300 nL/min, with a maximum pressure of 280 bar. The electrospray voltage was 1.8 kV and the temperature of the ion-transfer capillary was 300°C. The survey scan was set in the range of *m/z* values 390–2000 MS/MS. Each survey scan (MS) was followed by automated sequential selection of seven peptides for CID, with dynamic exclusion of the previously selected ions.

### Protein identification

The acquired MS/MS spectra generated were searched against Human protein database (Swiss PROT and TREMBL, http://ca.expasy.org) using Proteome Discoverer 1.3 software and SEQUEST algorithm. Parameter Xcorr was used. Search results were filtered for a False Discovery Rate of 1%, employing a decoy search strategy utilizing a reverse database. Each sample was analyzed four consecutive times by the mass spectrometer. For positive identification, the same protein had to be identified in at least three runs.

### Bioinformatics

Proteins identified for each surface group were compared using the Venny 2.1 online tool [26]. The proteins were further classified and assigned by biological function, molecular weight, and isoelectric point (pI) using the Gene Ontology (GO) terms obtained from the Uniprot databases [27].

## Results

### Metallic bracket surface characterization

**Fig 2:**
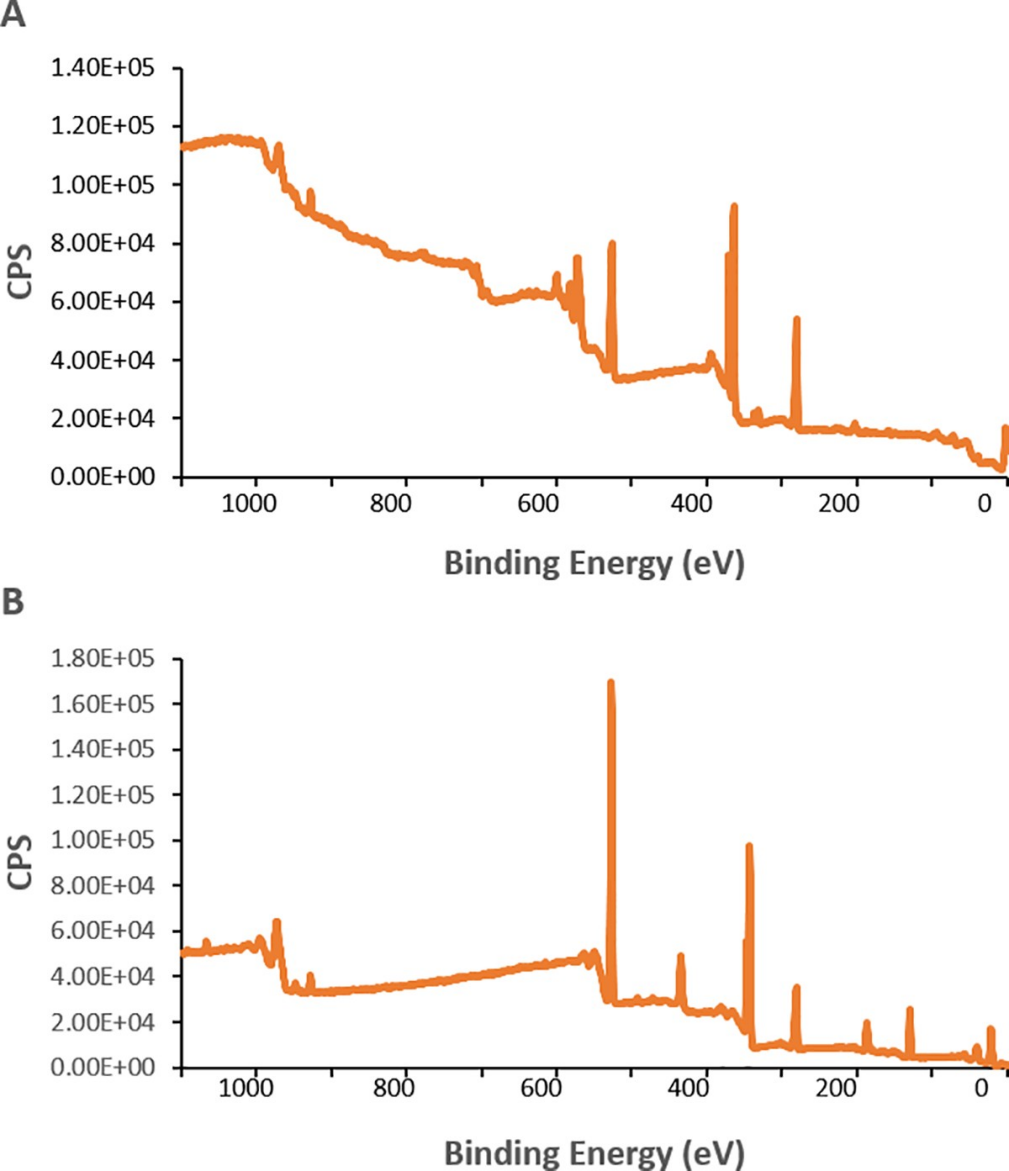
XPS wide scan spectrum for each specific surface: (A) metallic brackets, pre-treated with distilled water, and (B) HA discs, pre-treated with distilled water.

Results from the XPS analysis through wide scan spectrum of the metallic brackets indicated the presence of iron (Fe), silicon (Si), chromium (Cr), copper (Cu), sodium (Na), boron (B), nitrogen (N), sulfur (S), niobium (Nb), and palladium (Pd) (Fig 2). The wide scan spectrum of the control specimen (HA discs) show the presence of calcium (342 eV) and phosphorus (128 eV) as major components. Atoms % of calcium and phosphorus ratio (Ca/P ratio) was calculated as 1.45. The mean and standard derivation of the atomic concentration in both groups was presented in Table 1.

**Table 1:** Percentage of atoms (%) of on the surface of metallic brackets (MB) and hydroxyapatite discs (HA) incubated in distilled water (Mean ± S.D.).

MB		HAD	
Atom	%	Atom	%
**Ag**	10.6 ± 0.8	**I**	0.1 ± 0.0
**B**	5.3 ± 0.4	**Ca**	21.4 ± 0.2
**Cr**	12.8 ± 1.3	**Cu**	0.8 ± 0.1
**Cu**	1.6 ± 0.2	**F**	0.2 ± 0.3
**Fe**	3.4 ± 0.7	**Na**	0.5 ± 0.4
**N**	5.9 ± 1.1	**O**	62.4 ± 0.5
**Na**	0.7 ± 0.5	**P**	14.7 ± 0.8
**Nb**	1.5 ± 0.2		
**O**	55.0 ± 0.4		
**Pd**	0.9 ± 0.0		
**S**	1.1 ± 0.3		
**Si**	1.2 ± 0.4		

### Metallic bracket proteome identification and characterization

**Fig 3:**
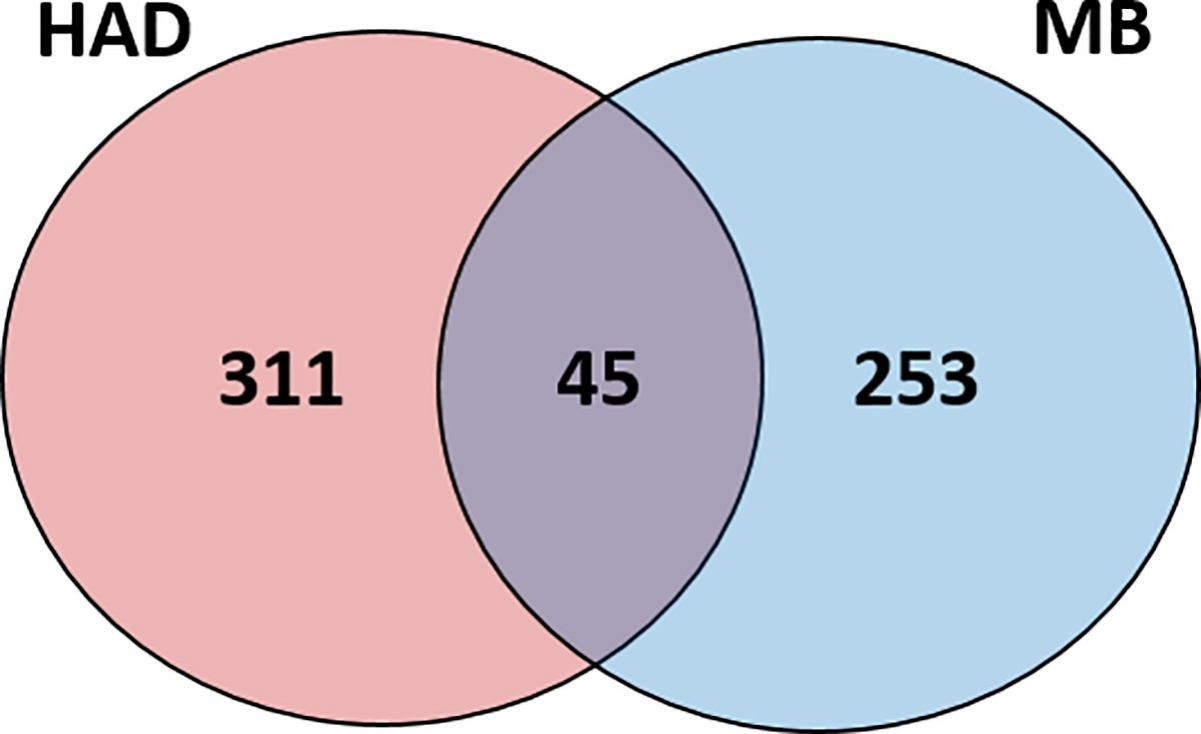
Venn diagram of acquired pellicle proteins identified in each study group and across groups. MB: metallic bracket, HAD: HA discs.

A total of 356 proteins were identified in the pellicle harvested from the HA disc control group (HA), and 298 proteins from the metallic orthodontic brackets (MB) (S1 Table). Forty-five proteins were common to the two groups, 311 proteins were unique to the HA discs, and 253 proteins were unique to the metallic brackets (Table 2). As expected, most of these proteins were identified specifically in one group, indicating little overlapping between the proteins from each group (Fig 3).

**Table 2:** Proteins identified in both groups, hydroxyapatite discs and metallic orthodontic bracket.

MB/HAD	Accession Number	Description (protein name)	Peptides Identification	MW [kDa]	calc. pI
Total: 45	P02814	Submaxillary gland androgen-regulated protein 3B	2	8.2	9.57
	F5GZK2	Collagen alpha-1(XXI)	3	99.2	8.47
	Q8WXI7	Mucin-16	14	1518.2	5.26
	E9PAV3	Nascent polypeptide-associated complex subunit alpha	6	205.3	9.58
	Q7Z5P9	Mucin-19	7	804.8	5.01
	Q5VST9	Obscurin	3	867.9	5.99
	Q9NR48	Histone-lysine N-methyltransferase ASH1L	3	332.6	9.39
	Q8IVF2	Protein AHNAK2	3	616.2	5.36
	C9JFF0	Kinesin-like protein KIF26A	2	180	8.9
	P15515	Histatin-1	2	7	9.14
	A2A2V2	RNA-binding protein 34	2	45.9	10.08
	T1R7N3	MUC5AC	4	413.6	8.09
	Q5SYE7	NHS-like protein 1	4	170.6	6.96
	Q86YZ3	Hornerin	4	282.2	10.04
	B8XCX8	EPC1/ASXL2b fusion protein	5	201.7	8.53
	B4DH81	cDNA FLJ61250	2	93.5	6.92
	P51826	AF4/FMR2 family member 3	3	133.4	8.1
	Q8IVL0	Neuron navigator 3	4	255.5	8.76
	Q6ZU65	Ubinuclein-2	3	146	9.19
	O75592	E3 ubiquitin-protein ligase MYCBP2	4	509.8	7.03
	A0A1W2PR28	IQ motif and SEC7 domain-containing protein 2	3	127.9	6.96
	Q96JG9	Zinc finger protein 469	3	409.9	7.72
	Q5T4S7	E3 ubiquitin-protein ligase UBR4	3	573.5	6.04
	X6R7H2	Peroxisome proliferator-activated receptor gamma coactivator-related protein 1	3	69.1	10.04
	Q96F05	Uncharacterized protein C11orf24	2	46.1	5.87
	Q59G99	Dishevelled 1 isoform a variant	2	40.3	8.24
	E7EPM4	Mucin-17	3	425.3	4.03
	P46013	Proliferation marker protein Ki-67	3	358.5	9.45
	Q9HCK8	Chromodomain-helicase-DNA-binding protein 8	2	290.3	6.47
	Q5VWG9	Transcription initiation factor TFIID subunit 3	3	103.5	9.06
	P25054	Adenomatous polyposis coli protein	3	311.5	7.8
	A0A140VJJ5	Testicular tissue protein Li 69	2	119.7	9.39
	Q9UKN1	Mucin-12	2	557.8	5.55
	O60307	Microtubule-associated serine/threonine-protein kinase 3	2	143	8.06
	E9PL24	Myomegalin	2	126.9	5.27
	Q96KW2	POM121-like protein 2	2	109.8	9.89
	L8E9Z3	Alternative protein HRC	2	34.3	11.99
	Q9C0D2	Centrosomal protein of 295 kDa	2	295	6
	B2RWP0	Signal-induced proliferation-associated 1 like 3	2	194.5	8.32
	A0A024RDF7	Uncharacterized protein	2	194.5	7.8
	Q5T1R4	Transcription factor HIVEP3	2	259.3	7.81
	B7Z7H2	cDNA FLJ58079	2	106.3	8.44
	J3KNQ2	Fibronectin type III domain-containing protein 1	6	194.4	9.2
	P01036	Cystatin-S	3	16.2	5

**Fig 4:**
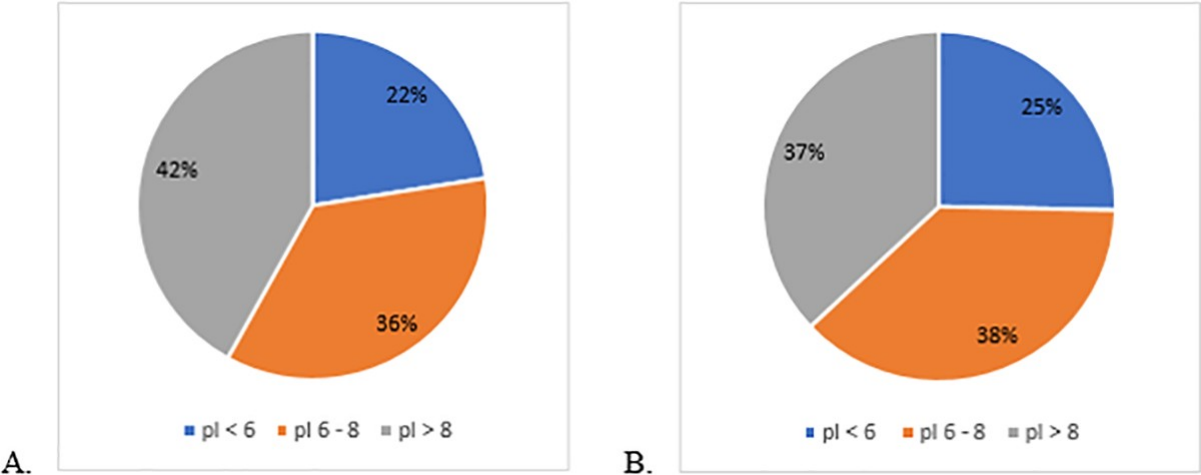
Distribution of proteins identified in each study group according to their isoelectric points: (A) metallic brackets, and (B) hydroxyapatite discs.

Based on the isoelectric point (pI) of the identified proteins, the metallic bracket pellicle had 67 proteins with pI below 6, 106 proteins had pI between 6–8, and 125 proteins had pI above 8. For the HA discs, 66 proteins had pI below 6, 128 had pI between 6–8, and 162 had pI above 8 (Fig 4).

According to biological functions (Table 3), proteins responsible for antimicrobial properties and/or involved on enamel demineralization/remineralization processes were found in both groups, exemplified by mucins and histatin 1, respectively. Moreover, amylase, a protein responsible for early plaque formation, was present on HA discs, but it was not found on metallic brackets.

**Table 3:** Proteins identified in the pellicle formed on metallic brackets and on hydroxyapatite discs categorized by biological function related to dental caries.

Biological Function	Metallic Brackets	Hydroxyapatite Discs
Antimicrobial	Mucin-16	Mucin-16
	Mucin-19	Mucin-19
	Histatin-1	Histatin-1
	MUC-5AC	MUC5AC
	Mucin-17	Mucin-17
	Mucin-12	Mucin-12
		Mucin-7
		Cystatin-S
Lubrication	Mucin-16	Mucin-16
	Proteoglycan 4	
Remineralization	Histatin-1	Histatin-1
	Statherin	Proline-rich protein 36
Buffering	1-phosphatidylinositol 4,5-bisphosphate phosphodiesterase eta-1	
Early dental plaque formation		Alpha-amylase

## Discussion

Caries lesions around and under orthodontic brackets is a common occurrence during orthodontic treatment which may cause delays in the treatment, increased costs, and irreversible loss of tooth tissue [28]. The development of dental caries is extremely influenced by the characteristics of the protein pellicle formed on the tooth surface and the subsequent biofilm. Since the surface composition of different materials selectively regulates the protein binding to the material [29], this study aimed at investigating the atomic composition of the surface of metallic orthodontic brackets, and at characterizing the protein pellicle formed on the surface of the brackets. XPS analysis was performed to determine the surface atomic composition of metallic orthodontic brackets, followed by identification of the proteins present in the harvested pellicle with mass spectrometry.

Previous studies have shown that the overall net charge of orthodontic brackets is usually neutral [30]; however, specific areas of different materials may have a positive or negative charge [29]. Therefore, we were not surprised to see that the bracket surface attracted proteins with both positive and negative charges without preference for one over the other. This is reinforced by the distribution of the proteins identified in the pellicle of metallic brackets according to their isoelectric point (pI), where both positive and negative proteins were found (Fig 4). Moreover, the charge of a protein is not the only factor that affects its interaction with a given surface. The size, structure, stability and unfolding rate of a protein also participate in their binding capacity. Furthermore, the composition of the surfaces as well as the topography, hydrophobicity and heterogeneity of the material are also factors [29] that influence the protein binding which must be considered.

Interestingly, most proteins were identified specifically to each group, suggesting specificity of the protein’s adsorption in relation to the surface of the bracket. As a result, biofilm formation [25] and bacterial adhesion [29] are expected to be different among the tested surfaces. Knowing that HA discs mimic the surface of the tooth [25], the difference between the proteins identified on the surface of the HA discs and those identified on the surface of the bracket suggests that metallic brackets have unique protein-adsorption properties that differ from human tooth enamel. Furthermore, even though most proteins identified were unique to each surface material, proteins responsible for antimicrobial activity, lubrication and remineralization function are present in both groups (Table 3). However, amylase, a protein considered to be directly involved in dental plaque formation, was not found in the pellicle of metallic brackets (Table 3).

In fact, amylase was uniquely identified in the pellicle formed on the HA discs. A previous study suggested that salivary alpha-amylase binds very selectively and with high affinity to several streptococcal species [3]. This is likely to influence commensal bacterial colonization [4] and to increase the sucrase and transferase activities of *Streptococccus mutans* [5]. Furthermore, starch may intensify the adhesion of amylase-binding streptococci to dental pellicles and increase the formation of dental plaque [3]. Nonetheless, clean metallic brackets were used for incubation with human saliva in this in-vitro study. It is very likely that the in vivo reality would involve food accumulation on the brackets, including considerable presence of starch residues, a situation that may favor the adsorption of other salivary proteins, such as amylase, for example. Moreover, a previous study suggested that ceramic brackets show greater affinity for *S*. *mutans* when compared to metallic brackets [31], an observation that may result from differences in the characteristics of the protein pellicle formed on different materials.

Other proteins with high affinity for HA surfaces are cystatins [22]. While no cystatins were found on metallic brackets, cystatin S was identified in the pellicle of HA discs. Cystatin S is known to participate in the enamel pellicle formation and to inhibit HA crystal growth [32]. Cystatins are also known for their cysteine inhibition capacity. In fact, cystatin SN appears to be more effective against host cathepsins H and L [33], involved in the destruction of periodontal tissue [34]. Additionally, antimicrobial activity against oral pathogens involved in periodontal diseases such as *Aggregatibacter actinomycetemcomitans* has been suggested for cystatin SA [35].

On the other hand, histatin 1 was identified on both metallic brackets and HA discs. Histatin 1 possesses strong antifungal properties against *Candida albicans* [36]. In addition, modulation of mineral formation, antibacterial activity [37, 38] and protection against acid injury [39] have been reported as biological functions of histatin 1.

Surprisingly, although statherin is known for its strong affinity for the tooth surface, this protein was identified in our study in the pellicle formed on metallic brackets. Statherin shows critical function in tooth remineralization as it is the only salivary protein to inhibit both primary (spontaneous) and secondary (crystal growth) calcium phosphate precipitation [40]; thus, maintaining saliva supersaturated with calcium phosphate.

Different mucins were found in both groups. Mucins are proteins with high carbohydrate content. Overall, mucins play a role in lubrication, non-immune protection and they can modulate the adhesion of microorganisms to the oral tissue surfaces [41]. MUC5B and MUC7 are the major members of the salivary mucin family [41, 42]. MUC5B is a high-molecular-weight gel-forming mucin with affinity for HA [43] found in the AEP [35]. MUC7, on the other hand, is a low molecular weight monomeric mucin, with affinity for cementum [44]. MUC7 also binds to bacteria such as oral Streptococci [45]. High-molecular-weight mucins such as MUCs 16, 17, 19, 5AC, and 12 were identified on the surfaces of both groups. Furthermore, mucins can form complexes with other salivary proteins such as amylase [46], statherin, histatin and PRPs [43]; thus, facilitating their adhesion to the surface of different materials.

Besides confirming the expected differences in the molecular composition of metallic orthodontic brackets and HA discs, this study reaffirmed the selectivity in the binding of salivary proteins to surfaces in the oral cavity. Moreover, this first characterization of the pellicle formed on the surface of metallic brackets, suggests that the metallic surface attracts important calcium-binding proteins, such as histatin 1 and statherin, which can assist in the remineralization process, while lacking proteins, such as amylase, with higher potential to bind cariogenic pathogens. Further investigation is needed to verify the effect of the characterized bracket pellicle on bacterial adhesion. Our results support the idea of surface modulation as an important path to favor the formation of bracket pellicles enriched with proteins with selected biological functions to prevent dental caries.

## Conclusions

Selective adsorption of proteins is observed in many different surface materials. Similarly, the adsorption of salivary proteins to the surface of orthodontic metallic brackets happens in a selective fashion. Interestingly, proteins with recognized high cariogenic characteristics were not identified on the metallic bracket pellicle, suggesting a protein pellicle with lower cariogenic activity. Further improvement in oral health and reduction of caries activity during orthodontic treatment may be explored via additional modulation of the acquired bracket pellicle by bracket surface modifications.

## Supporting information

S1 Table Total pellicle proteins present on metallic brackets surface and hydroxyapatite discs. (DOCX)
