# Supplementary material for: Proteome difference among the salivary proteins adsorbed onto metallic orthodontic brackets and hydroxyapatite discs
Source: PLoS One. 2021 Jul 28;16(7):e0254909. doi: 10.1371/journal.pone.0254909 (PMC8318307; doi:10.1371/journal.pone.0254909)
Supplement: S1 Table — (DOCX) [file pone.0254909.s001.docx]

**S1 Table**. Total pellicle proteins present on metallic brackets surface and hydroxyapatite discs.

| **Metallic Brackets** | | | | |
| --- | --- | --- | --- | --- |
| Accession Number | Description (protein name) | | Ion count score | MW [kDa] |
| P13645 | Keratin, type I cytoskeletal 10 | | 263.77 | 58.79 |
| A0A0A0MTS7 | Titin | | 175.26 | 3992.17 |
| H6VRF8 | Keratin 1 | | 175.13 | 66.01 |
| P02814 | Submaxillary gland androgen-regulated protein 3B | | 105.99 | 8.18 |
| P35527 | Keratin, type I cytoskeletal 9 | | 96.87 | 62.03 |
| P35908 | Keratin, type II cytoskeletal 2 epidermal | | 96.10 | 65.39 |
| M0R088 | Serine/arginine repetitive matrix protein 1 | | 59.52 | 78.09 |
| Q6ZRI6 | Uncharacterized protein C15orf39 | | 56.38 | 110.60 |
| P15515 | Histatin-1 | | 54.31 | 6.96 |
| B3KXW2 | cDNA FLJ46178 fis, clone TESTI4003944 | | 50.78 | 141.62 |
| F5GZK2 | Collagen alpha-1(XXI) | | 49.95 | 99.23 |
| Q8WXI7 | Mucin-16 | | 47.89 | 1518.24 |
| Q96RT6 | cTAGE family member 2 | | 41.43 | 85.23 |
| B4DRU6 | cDNA FLJ54657, highly similar to Keratin, type II cytoskeletal 6A | | 39.88 | 58.50 |
| Q92954 | Proteoglycan 4 | | 39.63 | 150.98 |
| Q5CZC0 | Fibrous sheath-interacting protein 2 | | 34.44 | 780.12 |
| B4DVQ0 | cDNA FLJ58286, highly similar to Actin, cytoplasmic 2 | | 33.09 | 37.32 |
| A0A0J9YYJ7 | Unconventional myosin-XVB | | 32.86 | 79.47 |
| A2A2V2 | RNA-binding protein 34 | | 32.18 | 45.89 |
| B3KNX8 | cDNA FLJ30689 fis, clone FCBBF2000566 | | 30.34 | 84.45 |
| E9PAV3 | Nascent polypeptide-associated complex subunit alpha, muscle-specific form | | 30.12 | 205.29 |
| Q7Z5P9 | Mucin-19 | | 30.11 | 804.77 |
| Q6P4R9 | SASH1 protein | | 25.96 | 134.74 |
| Q01546 | Keratin, type II cytoskeletal 2 oral | | 25.07 | 65.80 |
| Q9C0F0 | Putative Polycomb group protein ASXL3 | | 22.60 | 241.77 |
| P02808 | Statherin | | 22.41 | 7.30 |
| O60382 | KIAA0324 | | 22.20 | 191.19 |
| A0A0U1RRH1 | Ryanodine receptor 3 | | 20.06 | 550.47 |
| Q3SY84 | Keratin, type II cytoskeletal 71 | | 19.15 | 57.26 |
| Q5T3J3 | Ligand-dependent nuclear receptor-interacting factor 1 | | 17.82 | 84.52 |
| A8K781 | cDNA FLJ75299 | | 17.80 | 47.29 |
| T1R7N3 | MUC5AC | | 16.90 | 413.62 |
| Q5VST9 | Obscurin | | 16.86 | 867.94 |
| Q5SYE7 | NHS-like protein 1 | | 16.58 | 170.56 |
| A0A024RBF1 | Protein phosphatase 1, regulatory (Inhibitor) subunit 12A, isoform CRA_a | | 16.55 | 101.54 |
| A4UGR9 | Xin actin-binding repeat-containing protein 2 | | 16.29 | 382.06 |
| Q86UP3 | Zinc finger homeobox protein 4 | | 16.13 | 393.48 |
| H7BY37 | Histone-lysine N-methyltransferase 2C | | 16.09 | 269.44 |
| Q2KHM5 | CENPJ protein | | 16.02 | 129.87 |
| G8GEI5 | Coagulation factor VIII | | 15.77 | 8.36 |
| B7ZA42 | cDNA, FLJ79056 | | 15.51 | 77.19 |
| Q9NSI6 | Bromodomain and WD repeat-containing protein 1 | | 15.39 | 262.77 |
| Q9ULU4 | Protein kinase C-binding protein 1 | | 15.27 | 131.61 |
| K7ERE3 | Keratin, type I cytoskeletal 13 | | 15.18 | 45.23 |
| C9JE98 | Nuclear receptor corepressor 2 | | 15.05 | 267.88 |
| Q86YZ3 | Hornerin | | 14.75 | 282.23 |
| A0A0B4J1Z0 | COBL-like 1, isoform CRA_a | | 14.40 | 46.75 |
| A0A087WZ32 | Pleckstrin homology domain-containing family A member 2 | | 14.39 | 41.18 |
| B8XCX8 | EPC1/ASXL2b fusion protein | | 13.90 | 201.73 |
| B4DDN6 | cDNA FLJ54422 | | 13.75 | 71.96 |
| E9PPJ1 | Kinetochore scaffold 1 | | 13.69 | 195.52 |
| Q9UJT9 | F-box/LRR-repeat protein 7 | | 13.55 | 54.54 |
| A0A1U9XBF2 | MDC1 | | 13.35 | 226.47 |
| B4E1A2 | Gamma-aminobutyric acid (GABA) A receptor, alpha 5, isoform CRA_a | | 13.31 | 38.99 |
| P54259 | Atrophin-1 | | 13.22 | 125.34 |
| Q8WXG9 | G-protein coupled receptor 98 | | 13.19 | 692.64 |
| B4DH81 | cDNA FLJ61250 | | 13.18 | 93.45 |
| Q96HA1 | Nuclear envelope pore membrane protein POM 121 | | 13.17 | 127.64 |
| P51826 | AF4/FMR2 family member 3 | | 13.15 | 133.39 |
| A0A1U9XBE1 | MDC1 | | 13.06 | 226.46 |
| A0A1B0GWE0 | Dedicator of cytokinesis protein 7 (Fragment) | | 13.05 | 139.52 |
| Q66PJ3 | ADP-ribosylation factor-like protein 6-interacting protein 4 | | 12.93 | 44.89 |
| Q15751 | Probable E3 ubiquitin-protein ligase HERC1 | | 12.90 | 531.89 |
| Q9NXV6 | CDKN2A-interacting protein | | 12.83 | 61.09 |
| Q8N7X4 | Melanoma-associated antigen B6 | | 12.62 | 43.96 |
| A0A087WW76 | Rho GTPase-activating protein 21 | | 12.42 | 155.89 |
| B5M450 | Anion exchange protein | | 12.40 | 135.22 |
| Q8IVL0 | Neuron navigator 3 | | 12.32 | 255.49 |
| Q6LEQ8 | LTG9/MLLT3 protein | | 11.59 | 38.09 |
| Q96T58 | Msx2-interacting protein | | 11.45 | 402.00 |
| Q6ZU65 | Ubinuclein-2 | | 11.33 | 146.00 |
| J3KQ96 | Treacle protein | | 11.25 | 144.04 |
| Q6PII6 | TMF1 protein | | 11.25 | 58.35 |
| A0A1U9X7B3 | BRD2 | | 11.21 | 74.71 |
| Q7Z7G8 | Vacuolar protein sorting-associated protein 13B | | 11.11 | 448.38 |
| O75592 | E3 ubiquitin-protein ligase MYCBP2 | | 11.08 | 509.76 |
| B2RAG9 | Mediator of RNA polymerase II transcription subunit 1 | | 11.08 | 166.54 |
| Q9UPN3 | Microtubule-actin cross-linking factor 1, isoforms 1/2/3/5 | | 11.06 | 837.79 |
| Q5KU20 | G-protein coupled receptor | | 11.05 | 102.05 |
| Q6N055 | Putative uncharacterized protein DKFZp686O11112 | | 11.03 | 35.54 |
| H3BM45 | Ankyrin repeat and fibronectin type-III domain-containing protein 1 | | 10.87 | 129.87 |
| Q59H94 | Gamma filamin variant | | 10.69 | 142.73 |
| F4MH51 | Ubiquitously transcribed tetratricopeptide repeat protein Y-linked transcript variant 60 | | 10.41 | 147.07 |
| D6RD46 | LIM and calponin homology domains-containing protein 1 | | 10.35 | 109.87 |
| B7Z2P9 | E3 ubiquitin-protein ligase | | 10.34 | 96.34 |
| A0A1W2PR28 | IQ motif and SEC7 domain-containing protein 2 | | 10.31 | 127.90 |
| Q96JG9 | Zinc finger protein 469 | | 10.29 | 409.95 |
| Q9BTM2 | C2CD2L protein | | 10.17 | 28.22 |
| Q9NZR2 | Low-density lipoprotein receptor-related protein 1B | | 10.17 | 515.16 |
| O95996 | Adenomatous polyposis coli protein 2 | | 10.09 | 243.80 |
| B4DJT3 | cDNA FLJ54498, weakly similar to Mucin-like protein 1 | | 10.04 | 67.74 |
| A0A024RAN2 | Calpastatin, isoform CRA_a | | 10.03 | 86.56 |
| B7ZLW1 | CAMSAP1 protein | | 10.01 | 163.16 |
| Q5T4S7 | E3 ubiquitin-protein ligase UBR4 | | 9.99 | 573.48 |
| F8VZ81 | TBC1 domain family member 30 | | 9.99 | 71.78 |
| X6R7H2 | Peroxisome proliferator-activated receptor gamma coactivator-related protein 1 | | 9.98 | 69.06 |
| Q53F67 | Kringle-containing transmembrane protein 2 isoform b variant | | 9.96 | 44.42 |
| Q6ZS56 | cDNA FLJ45819 fis, clone NT2RP8001363 | | 9.96 | 66.96 |
| Q96F05 | Uncharacterized protein C11orf24 | | 9.93 | 46.07 |
| Q8IVF2 | Protein AHNAK2 | | 9.91 | 616.24 |
| A0A087WXN9 | MTSS1-like protein | | 9.90 | 79.75 |
| Q9UL49 | Transcription factor-like 5 protein | | 9.89 | 52.66 |
| H0YLN8 | Transient receptor potential cation channel subfamily M member 7 | | 9.87 | 212.49 |
| B3KPL9 | cDNA FLJ31930 fis, clone NT2RP7006162 | | 9.85 | 47.82 |
| A0A0G2JN73 | Diffuse panbronchiolitis critical region protein 1 | | 9.84 | 56.31 |
| Q05CT8 | NEDD1 protein | | 9.83 | 57.47 |
| A0A1B0GUS7 | Protein unc-13 homolog B | | 9.79 | 483.83 |
| Q9NR48 | Histone-lysine N-methyltransferase ASH1L | | 9.76 | 332.58 |
| Q53QN9 | Putative uncharacterized protein ABCG5 | | 9.76 | 60.84 |
| A0A1B0GV45 | Unconventional myosin-XVIIIa | | 9.74 | 49.69 |
| I0B0K8 | Truncated profilaggrin | | 9.72 | 430.16 |
| B7Z526 | cDNA FLJ59292 | | 9.71 | 85.62 |
| H7C1L9 | E3 ubiquitin-protein ligase TRIP12 | | 9.69 | 22.41 |
| Q59G99 | Dishevelled 1 isoform a variant | | 9.69 | 40.28 |
| E7EPM4 | Mucin-17 | | 9.64 | 425.29 |
| H3BR17 | E3 ubiquitin-protein ligase TRAF7 | | 9.63 | 15.86 |
| B4E392 | cDNA FLJ52602, highly similar to Pinin | | 9.63 | 49.11 |
| Q9UHR4 | Brain-specific angiogenesis inhibitor 1-associated protein 2-like protein 1 | | 9.63 | 56.85 |
| Q92610 | Zinc finger protein 592 | | 9.62 | 137.44 |
| A0A1W2PQJ7 | Retinoic acid-induced protein 1 | | 9.54 | 183.82 |
| P46013 | Proliferation marker protein Ki-67 | | 9.54 | 358.47 |
| K7EMR9 | Dynamin-2 | | 9.53 | 18.20 |
| Q9HCK8 | Chromodomain-helicase-DNA-binding protein 8 | | 9.52 | 290.34 |
| F5H101 | Nucleolar protein 8 | | 9.48 | 121.52 |
| P56555 | Down syndrome critical region protein 4 | | 9.37 | 12.95 |
| Q8NA90 | cDNA FLJ35733 fis, clone TESTI2003277 | | 9.36 | 29.45 |
| A8KAI3 | cDNA FLJ77394 | | 9.09 | 57.44 |
| H3BLS7 | Vacuolar protein sorting-associated protein 13D | | 8.99 | 356.81 |
| A0A024QZW7 | Nucleoporin 153kDa, isoform CRA_a | | 8.92 | 153.87 |
| B3KR92 | cDNA FLJ33882 fis, clone CTONG2007175 | | 8.84 | 61.73 |
| A8K8M7 | cDNA FLJ76439 | | 8.82 | 53.73 |
| Q5VWG9 | Transcription initiation factor TFIID subunit 3 | | 8.76 | 103.52 |
| Q14686 | Nuclear receptor coactivator 6 | | 8.73 | 219.01 |
| A0A1W2PQW2 | Voltage-dependent T-type calcium channel subunit alpha-1H | | 8.59 | 178.43 |
| Q9UPU5 | Ubiquitin carboxyl-terminal hydrolase 24 | | 8.53 | 294.18 |
| P13611 | Versican core protein | | 8.46 | 372.59 |
| P25054 | Adenomatous polyposis coli protein | | 8.36 | 311.45 |
| Q02388 | Collagen alpha-1(VII) | | 8.32 | 295.04 |
| A0A0A0MS79 | Nck-associated protein 5 | | 8.26 | 208.35 |
| Q9UIF8 | Bromodomain adjacent to zinc finger domain protein 2B | | 8.26 | 240.31 |
| A0A140VJJ5 | Testicular tissue protein Li 69 | | 8.13 | 119.70 |
| Q8WVS4 | WD repeat-containing protein 60 | | 8.07 | 122.50 |
| A0A0U1RQK4 | [Protein ADP-ribosylarginine] hydrolase-like protein 1 | | 7.97 | 211.69 |
| Q8NB66 | Protein unc-13 homolog C | | 7.86 | 250.75 |
| Q7Z2Y8 | Interferon-induced very large GTPase 1 | | 7.83 | 278.87 |
| F8WDA1 | 5'-AMP-activated protein kinase subunit gamma-2 | | 7.80 | 24.62 |
| B5A965 | Soluble FGFR4 variant 2 | | 7.48 | 48.62 |
| H7BY35 | Ryanodine receptor 2 | | 7.37 | 562.25 |
| Q9UKN1 | Mucin-12 | | 7.25 | 557.83 |
| A0A1S5UZ29 | Kalirin | | 7.24 | 269.17 |
| H0YL16 | Serine/threonine-protein kinase PAK 6 | | 7.21 | 30.72 |
| Q86XZ4 | Spermatogenesis-associated serine-rich protein 2 | | 7.20 | 59.51 |
| Q5T0Z8 | Uncharacterized protein C6orf132 | | 7.18 | 123.96 |
| A8K8U1 | cDNA FLJ77762 | | 7.17 | 136.23 |
| Q9NYB5 | Solute carrier organic anion transporter family member 1C1 | | 7.15 | 78.65 |
| D3DWL9 | Cleavage and polyadenylation specific factor 1, 160kDa, isoform CRA_a | | 7.11 | 151.89 |
| Q9NT22 | EMILIN-3 | | 7.08 | 82.60 |
| B1WB49 | BDP1 protein | | 7.04 | 293.25 |
| Q6WKZ4 | Rab11 family-interacting protein 1 | | 6.96 | 137.08 |
| Q8WYX4 | Putative uncharacterized protein pp11662 | | 6.94 | 21.03 |
| P49916 | DNA ligase 3 | | 6.94 | 112.83 |
| O60307 | Microtubule-associated serine/threonine-protein kinase 3 | | 6.93 | 143.05 |
| E9PL24 | Myomegalin | | 6.88 | 126.87 |
| H7BZB4 | C2 domain-containing protein 3 | | 6.88 | 86.23 |
| B4E345 | cDNA FLJ50374, weakly similar to Protein groucho | | 6.86 | 61.71 |
| Q9UF83 | Uncharacterized protein DKFZp434B061 | | 6.86 | 59.38 |
| D3DR86 | Nuclear factor of kappa light polypeptide gene enhancer in B-cells 2 (P49/p100) | | 6.84 | 44.23 |
| Q5M9Q1 | NKAP-like protein | | 6.83 | 46.28 |
| Q9Y5S2 | Serine/threonine-protein kinase MRCK beta | | 6.83 | 194.19 |
| A2SY06 | MAP/microtubule affinity-regulating kinase 3 | | 6.81 | 36.68 |
| Q9NRK6 | ATP-binding cassette sub-family B member 10, mitochondrial | | 6.80 | 79.10 |
| P98169 | Zinc finger X-linked protein ZXDB | | 6.80 | 84.74 |
| A0A075B6G6 | Filamin A interacting protein 1, isoform CRA_c | | 6.80 | 127.13 |
| Q9HCM3 | UPF0606 protein KIAA1549 | | 6.80 | 210.63 |
| Q969K3 | E3 ubiquitin-protein ligase RNF34 | | 6.80 | 41.61 |
| H0YC33 | La-related protein 1 | | 6.79 | 20.88 |
| B9ZVN9 | DNA-directed RNA polymerase subunit | | 6.78 | 187.69 |
| A8KAY2 | Fibrillin 3 | | 6.76 | 300.12 |
| B4E223 | cDNA FLJ52075 | | 6.76 | 68.46 |
| Q6ZMZ1 | cDNA FLJ16568 fis, clone TESOP2000390 | | 6.74 | 52.00 |
| C9J164 | Ras-associated and pleckstrin homology domains-containing protein 1 | | 6.72 | 67.14 |
| A0A024R7D8 | Ral guanine nucleotide dissociation stimulator-like 3, isoform CRA_a | | 6.72 | 51.08 |
| Q8NAV8 | cDNA FLJ34691 fis, clone MESAN2000909 | | 6.72 | 64.30 |
| B4DGV8 | cDNA FLJ54286 | | 6.71 | 19.45 |
| H7C189 | Ubiquitin carboxyl-terminal hydrolase 4 | | 6.70 | 69.51 |
| Q8N0Z3 | Spindle and centriole-associated protein 1 | | 6.69 | 96.21 |
| Q9UJ55 | MAGE-like protein 2 | | 6.69 | 132.74 |
| Q96KW2 | POM121-like protein 2 | | 6.69 | 109.84 |
| Q5T1H1 | Protein eyes shut homolog | | 6.68 | 350.57 |
| A8MXZ3 | Keratin-associated protein 9-1 | | 6.68 | 26.31 |
| Q6DKI7 | Transmembrane protein PVRIG | | 6.68 | 34.32 |
| P47989 | Xanthine dehydrogenase/oxidase | | 6.67 | 146.33 |
| B2RTX2 | Palladin, cytoskeletal associated protein | | 6.67 | 121.97 |
| A0A0A0MS59 | Helicase SRCAP | | 6.66 | 315.42 |
| A0A090N8E5 | Similar to OG-2 homeodomain protein-like similar to U65067 (PID:g1575526) | | 6.65 | 52.71 |
| Q0IIP3 | C20orf194 protein | | 6.65 | 102.18 |
| A0A024RAL3 | Zinc finger, FYVE domain containing 16, isoform CRA_a | | 6.65 | 168.74 |
| M0R219 | Liprin-alpha-4 | | 6.64 | 20.44 |
| Q53TT7 | Putative uncharacterized protein ALS2CR3 | | 6.63 | 97.93 |
| L8E9Z3 | Alternative protein HRC | | 6.62 | 34.32 |
| O43149 | Zinc finger ZZ-type and EF-hand domain-containing protein 1 | | 6.62 | 330.86 |
| Q6ZWP8 | Inactive rhomboid protein | | 6.62 | 80.36 |
| M0R0C4 | Dystrobrevin alpha | | 6.60 | 18.64 |
| A0A5B4 | T-cell receptor beta variable 20-1 | | 6.60 | 12.20 |
| V9GZ26 | Protein FAM110A | | 6.60 | 19.68 |
| M0R0F4 | Atypical kinase COQ8B, mitochondrial | | 6.60 | 12.11 |
| J3QKX6 | SWI/SNF-related matrix-associated actin-dependent regulator of chromatin subfamily E member 1 | | 6.60 | 14.60 |
| P35900 | Keratin, type I cytoskeletal 20 | | 6.59 | 48.46 |
| A0A1S5UYZ7 | Rap guanine nucleotide exchange factor 1 | | 6.56 | 120.16 |
| Q13233 | Mitogen-activated protein kinase kinase kinase 1 | | 6.56 | 164.37 |
| Q8IYJ2 | Uncharacterized protein C10orf67, mitochondrial | | 6.55 | 63.62 |
| B2RB68 | cDNA, FLJ95336 | | 6.55 | 67.80 |
| B4DQR1 | cDNA FLJ55241 | | 6.55 | 22.93 |
| Q4EW05 | Rhesus blood group D antigen | | 6.55 | 6.03 |
| B4DLJ7 | cDNA FLJ59334 | | 6.54 | 57.18 |
| A0A087WUH9 | Serine/threonine-protein kinase PLK | | 6.53 | 76.60 |
| Q9ULL0 | Acrosomal protein KIAA1210 | | 6.52 | 186.90 |
| B3KVV3 | cDNA FLJ41584 fis, clone CTONG2020445 | | 6.52 | 72.29 |
| E9PCY0 | Dynactin subunit 1 | | 6.52 | 19.44 |
| Q9Y4F1 | FERM, RhoGEF and pleckstrin domain-containing protein 1 | | 6.52 | 118.56 |
| A0A024R2Y4 | Bassoon (Presynaptic cytomatrix protein), isoform CRA_a | | 6.52 | 416.24 |
| Q86YS7 | C2 domain-containing protein 5 | | 6.50 | 110.38 |
| D3DPE6 | Wiskott-Aldrich syndrome protein interacting protein, isoform CRA_a | | 6.49 | 50.27 |
| O60293 | Zinc finger C3H1 domain-containing protein | | 6.48 | 226.21 |
| H3BN61 | Doublesex- and mab-3-related transcription factor 1 | | 6.48 | 23.16 |
| P39880 | Homeobox protein cut-like 1 | | 6.47 | 164.09 |
| J3KSW8 | Myosin phosphatase Rho-interacting protein | | 6.45 | 95.87 |
| H0Y797 | Thyrotroph embryonic factor | | 6.45 | 20.00 |
| A5XEH6 | WNK lysine deficient protein kinase 1 | | 6.44 | 7.71 |
| E9PM59 | TATA box-binding protein-associated factor RNA polymerase I subunit D | | 6.44 | 25.03 |
| H0YEU1 | CD44 antigen | | 6.43 | 27.26 |
| C9JR56 | DNA-binding protein SATB | | 6.43 | 75.99 |
| H0YGW3 | Protein FAM13C | | 6.43 | 15.38 |
| Q96RV3 | Pecanex-like protein 1 | | 6.43 | 258.51 |
| Q4KWH8 | 1-phosphatidylinositol 4,5-bisphosphate phosphodiesterase eta-1 | | 6.43 | 189.10 |
| J3KNV1 | Zinc finger protein 292 | | 6.43 | 304.02 |
| L8E767 | Alternative protein RNF222 | | 6.42 | 19.45 |
| F5GX59 | Zonadhesin | | 6.41 | 282.23 |
| F5H514 | RAS guanyl-releasing protein 1 | | 6.41 | 90.31 |
| O75113 | NEDD4-binding protein 1 | | 6.40 | 100.32 |
| C9JFF0 | Kinesin-like protein KIF26A | | 6.40 | 180.04 |
| Q59FH1 | Transformation/transcription domain-associated protein variant | | 6.39 | 405.56 |
| H0Y8C9 | ATP-binding cassette sub-family A member 2 | | 6.38 | 118.05 |
| A0A087WYF1 | Laminin subunit alpha-2 | | 6.38 | 343.20 |
| A9UF07 | BCR/ABL fusion protein isoform Y5 | | 6.37 | 196.45 |
| B4DHI4 | cDNA FLJ60536, highly similar to Death-associated protein kinase 1 (EC 2.7.11.1) OS=Homo sapiens PE=2 SV=1 - [B4DHI4_HUMAN] | | 6.36 | 159.96 |
| D3DSU3 | Kinesin family member 13B, isoform CRA_a | | 6.36 | 174.69 |
| F5H3X8 | Poly [ADP-ribose] polymerase | | 6.36 | 55.11 |
| Q53F19 | Nuclear cap-binding protein subunit 3 | | 6.35 | 70.55 |
| Q6R743 | MHC class I antigen | | 6.34 | 41.60 |
| P98088 | Mucin-5AC | | 6.34 | 585.20 |
| Q7LGH1 | KIAA0480 protein | | 6.32 | 146.90 |
| B5BUB1 | RuvB-like helicase | | 6.31 | 50.18 |
| Q9NX53 | Exonuclease mut-7 homolog, isoform 5 | | 6.29 | 26.34 |
| Q59F85 | Glucose phosphate isomerase variant | | 6.28 | 55.65 |
| Q53GZ4 | Leucine rich repeat containing 5 variant | | 6.26 | 76.80 |
| P61129 | Zinc finger CCCH domain-containing protein 6 | | 6.26 | 131.59 |
| B4E1I2 | cDNA FLJ61037, highly similar to Intraflagellar transport 88 homolog | | 6.26 | 12.38 |
| Q8N3N3 | Putative uncharacterized protein DKFZp762L056 | | 6.26 | 89.64 |
| Q59EC9 | Glyceronephosphate O-acyltransferase variant | | 6.25 | 78.54 |
| L8ECB8 | Alternative protein NR5A1 | | 6.25 | 19.48 |
| Q9C0D2 | Centrosomal protein of 295 kDa | | 6.22 | 295.00 |
| Q0PNF2 | FEX1 | | 6.14 | 275.27 |
| A6NCG2 | Solute carrier family 22 member 11 | | 6.13 | 52.58 |
| D6RGF0 | Protein Largen | | 6.12 | 15.22 |
| B0I1S4 | DNHD1 variant protein | | 6.08 | 428.42 |
| Q8IWQ8 | SCAMPER | | 6.07 | 12.41 |
| Q5TIG5 | Afadin | | 6.05 | 189.04 |
| Q05BX6 | RABEP1 protein | | 6.04 | 82.33 |
| Q8N3K9 | Cardiomyopathy-associated protein 5 | | 6.02 | 448.94 |
| A0A024RDF7 | Uncharacterized protein | | 5.99 | 130.17 |
| O00757 | Fructose-1,6-bisphosphatase isozyme 2 | | 5.98 | 36.72 |
| H0YC63 | Microtubule-associated tumor suppressor 1 | | 5.94 | 55.14 |
| I3L3K8 | Sodium-independent sulfate anion transporter | | 5.89 | 7.91 |
| Q9HBD1 | Roquin-2 | | 5.88 | 131.59 |
| B3KUX0 | cDNA FLJ40831 fis, clone TRACH2012138 | | 5.88 | 35.87 |
| Q5JR59 | Microtubule-associated tumor suppressor candidate 2 | | 5.84 | 150.10 |
| P26378 | ELAV-like protein 4 | | 5.84 | 41.74 |
| C1KEQ3 | GLCCI1 | | 5.80 | 3.59 |
| B2RWP0 | Signal-induced proliferation-associated 1 like 3 | | 5.80 | 194.50 |
| A0A0A0MRJ3 | Neuron navigator 1 | | 5.80 | 197.28 |
| Q8IXV0 | HES1 protein | | 5.79 | 29.25 |
| A1L0S8 | CROCC protein | | 5.79 | 129.39 |
| Q5T1R4 | Transcription factor HIVEP3 | | 5.75 | 259.30 |
| B2RE19 | cDNA, FLJ96877 | | 5.75 | 70.92 |
| B4DSW4 | cDNA FLJ51541, moderately similar to Transcription factor Sp8 | | 5.69 | 16.42 |
| Q6ZMY0 | cDNA FLJ16598 fis, clone TESTI4006473, weakly similar to ATP-dependent RNA helicase A | | 5.67 | 150.88 |
| C9JMI8 | TBC1 domain family member 25 | | 5.65 | 24.95 |
| Q6UWX4 | HHIP-like protein 2 | | 5.02 | 80.73 |
| A4PB67 | YY1AP-related protein1 | | 4.98 | 248.19 |
| E9PG32 | Dynein heavy chain 12, axonemal | | 4.97 | 454.03 |
| Q6ZSX8 | cDNA FLJ45139 fis, clone BRAWH3039623 | | 4.92 | 15.46 |
| O15078 | Centrosomal protein of 290 kDa | | 4.91 | 290.21 |
| Q8NDM7 | Cilia- and flagella-associated protein 43 | | 4.86 | 191.86 |
| Q8N1H6 | cDNA FLJ40869 fis, clone TSTOM2000139 | | 4.79 | 53.78 |
| O15014 | Zinc finger protein 609 | | 4.78 | 151.10 |
| Q5TAX3 | Terminal uridylyltransferase 4 | | 4.31 | 185.05 |
| B7Z7H2 | cDNA FLJ58079, highly similar to Homo sapiens SH3 multiple domains 1 (SH3MD1), mRNA | | 4.04 | 106.32 |
| **Hydroxyapatite Disc** | | | | |
| Accession Number | | Description (protein name) | Ion count score | MW [kDa] |
| Q8WXI7 | | Mucin-16 | 82.02 | 1518.2 |
| P15515 | | Histatin-1 | 68.85 | 7.0 |
| B7ZMD7 | | Alpha-amylase | 66.23 | 57.7 |
| D3DPG0 | | Titin, isoform CRA_a | 65.09 | 3878.8 |
| P02814 | | Submaxillary gland androgen-regulated protein 3B | 61.25 | 8.2 |
| Q7Z5P9 | | Mucin-19 | 50.38 | 804.8 |
| B3KY63 | | cDNA FLJ16830 fis, clone UTERU3022536 | 43.22 | 215.1 |
| F5GZK2 | | Collagen alpha-1(XXI) chain | 37.05 | 99.2 |
| P01036 | | Cystatin-S | 33.12 | 16.2 |
| A0A0U1RR20 | | Proteoglycan 4 | 30.51 | 146.4 |
| Q86VQ1 | | Glucocorticoid-induced transcript 1 protein | 29.92 | 58.0 |
| E9PAV3 | | Nascent polypeptide-associated complex subunit alpha, muscle-specific form | 24.97 | 205.3 |
| Q5VST9 | | Obscurin | 22.78 | 867.9 |
| H0Y465 | | Neurofibromin | 20.65 | 281.0 |
| T1S9D5 | | MUC5AC | 20.41 | 521.3 |
| T1R7N3 | | MUC5AC | 20.12 | 413.6 |
| B4DH81 | | cDNA FLJ61250 | 19.95 | 93.5 |
| B3KY54 | | cDNA FLJ46886 fis, clone UTERU3016308 | 19.84 | 61.6 |
| Q5SYE7 | | NHS-like protein 1 | 19.49 | 170.6 |
| Q9UQ35 | | Serine/arginine repetitive matrix protein 2 | 19.23 | 299.4 |
| A7E2D6 | | NAV2 protein | 18.74 | 261.6 |
| F8W9J4 | | Dystonin | 18.06 | 847.4 |
| Q96Q06 | | Perilipin-4 | 18.01 | 134.3 |
| J3KNQ2 | | Fibronectin type III domain-containing protein 1 | 17.88 | 194.4 |
| Q15648 | | Mediator of RNA polymerase II transcription subunit 1 | 17.29 | 168.4 |
| Q8N500 | | Putative uncharacterized protein | 16.87 | 36.0 |
| Q8IWQ1 | | TGS2 | 16.82 | 60.0 |
| Q5T4S7 | | E3 ubiquitin-protein ligase UBR4 | 16.60 | 573.5 |
| Q8IVF2 | | Protein AHNAK2 | 16.59 | 616.2 |
| Q5VUA4 | | Zinc finger protein 318 | 16.47 | 251.0 |
| I6L894 | | Ankyrin-2 | 16.31 | 430.0 |
| Q03164 | | Histone-lysine N-methyltransferase 2A | 16.13 | 431.5 |
| Q13136 | | Liprin-alpha-1 | 15.76 | 135.7 |
| Q8IVL0 | | Neuron navigator 3 | 15.67 | 255.5 |
| B7ZA42 | | cDNA, FLJ79056 | 15.57 | 77.2 |
| Q96HP0 | | Dedicator of cytokinesis protein 6 | 15.46 | 229.4 |
| P25054 | | Adenomatous polyposis coli protein | 14.87 | 311.5 |
| A0A1B0GVP4 | | Ligand-dependent nuclear receptor corepressor-like protein | 14.77 | 211.4 |
| M0R2B3 | | Uncharacterized protein C19orf44 | 14.23 | 68.2 |
| Q5D862 | | Filaggrin-2 | 14.14 | 247.9 |
| O60307 | | Microtubule-associated serine/threonine-protein kinase 3 | 13.98 | 143.0 |
| Q96F05 | | Uncharacterized protein C11orf24 | 13.94 | 46.1 |
| C9JFF0 | | Kinesin-like protein KIF26A | 13.74 | 180.0 |
| Q9UKN1 | | Mucin-12 | 13.65 | 557.8 |
| C9K0E4 | | Syntaxin-binding protein 5-like | 13.62 | 111.2 |
| O75592 | | E3 ubiquitin-protein ligase MYCBP2 | 13.48 | 509.8 |
| L8E7G9 | | Alternative protein ZNF74 | 13.30 | 32.4 |
| A6NK89 | | Ras association domain-containing protein 10 | 13.28 | 56.9 |
| B7Z7S7 | | cDNA FLJ60964, weakly similar to Homo sapiens dentin sialophosphoprotein (DSPP), mRNA | 13.27 | 37.7 |
| B3KWI5 | | cDNA FLJ43124 fis, clone CTONG3004072, highly similar to Protein EMSY | 13.26 | 130.7 |
| E9PG32 | | Dynein heavy chain 12, axonemal | 13.24 | 454.0 |
| F5GYR0 | | Actin-binding LIM protein 2 | 13.13 | 71.3 |
| H0YN99 | | Pseudopodium-enriched atypical kinase 1 | 13.13 | 115.1 |
| Q9NR48 | | Histone-lysine N-methyltransferase ASH1L | 13.06 | 332.6 |
| Q9H5Y7 | | SLIT and NTRK-like protein 6 | 13.00 | 95.0 |
| Q8TE73 | | Dynein heavy chain 5, axonemal | 12.98 | 528.7 |
| A7E2F7 | | CAP-GLY domain containing linker protein 2 | 12.95 | 111.7 |
| Q5HYC2 | | Uncharacterized protein KIAA2026 | 12.85 | 227.9 |
| A0A087WV20 | | Alstrom syndrome protein 1 | 12.77 | 425.0 |
| Q5T1R4 | | Transcription factor HIVEP3 | 12.73 | 259.3 |
| A7Y9J9 | | Mucin 5AC, oligomeric mucus/gel-forming | 12.59 | 648.4 |
| Q96RK0 | | Protein capicua homolog | 12.58 | 163.7 |
| A0A0J9YY01 | | Unconventional myosin-XVB | 12.41 | 333.5 |
| Q9Y2F5 | | Little elongation complex subunit 1 | 12.35 | 247.7 |
| A0A087X1X8 | | Uncharacterized protein | 12.31 | 21.7 |
| A0A0A0MS59 | | Helicase SRCAP | 12.23 | 315.4 |
| A1L4H1 | | Soluble scavenger receptor cysteine-rich domain-containing protein SSC5D | 12.20 | 165.6 |
| H7BZX1 | | Sorbin and SH3 domain-containing protein 2 | 12.05 | 27.5 |
| S6C4Q9 | | IgG L chain | 12.01 | 22.8 |
| B4DWY3 | | cDNA FLJ56165, highly similar to RNA exonuclease 1 homolog (EC 3.1.-.-) | 11.79 | 58.3 |
| P54274 | | Telomeric repeat-binding factor | 11.68 | 50.2 |
| Q2TAZ0 | | Autophagy-related protein 2 homolog A | 11.55 | 212.7 |
| P78310 | | Coxsackievirus and adenovirus receptor | 11.50 | 40.0 |
| A6NM62 | | Leucine-rich repeat-containing protein 53 | 11.39 | 140.7 |
| Q96LR2 | | Leucine rich adaptor protein 1 | 11.24 | 25.8 |
| K9N2R0 | | Interleukin 15 receptor alpha isoform IC8 OS=Ho | 11.14 | 30.8 |
| Q9H2L7 | | DC33 | 11.06 | 29.5 |
| Q9UHB7 | | AF4/FMR2 family member 4 | 10.92 | 127.4 |
| Q92766 | | Ras-responsive element-binding protein 1 | 10.85 | 181.3 |
| Q59GL0 | | Rearranged L-myc fusion sequence variant | 10.64 | 184.6 |
| Q7Z4S6 | | Kinesin-like protein KIF21A | 10.60 | 187.1 |
| Q6GMQ3 | | PHC1 protein | 10.56 | 100.1 |
| Q6KC79 | | Nipped-B-like protein | 10.53 | 315.9 |
| A0A087WVZ6 | | Protein kinase C-binding protein 1 | 10.51 | 125.7 |
| A0A087WXN4 | | Integrator complex subunit 12 | 10.51 | 46.6 |
| Q6ZU65 | | Ubinuclein-2 | 10.41 | 146.0 |
| E7EWQ5 | | Microtubule-associated serine/threonine-protein kinase 4 | 10.40 | 266.0 |
| A0A075B756 | | Krueppel-like factor 14 | 10.39 | 33.1 |
| F1T0K4 | | DmX-like protein 1 | 10.38 | 318.4 |
| H3BTR6 | | RNA-binding protein with serine-rich domain 1 | 10.36 | 13.0 |
| Q2M2I8 | | AP2-associated protein kinase 1 | 10.28 | 103.8 |
| Q9BXF6 | | Rab11 family-interacting protein 5 | 10.26 | 70.4 |
| A8KAL3 | | cDNA FLJ77478, highly similar to Homo sapiens Rho GTPase activating protein 6 (ARHGAP6), transcript variant 1, mRNA | 10.24 | 105.8 |
| P78409 | | Butyrophilin | 10.20 | 81.3 |
| H7C1I7 | | Zinc finger MYM-type protein 4 | 10.19 | 134.3 |
| Q15772 | | Striated muscle preferentially expressed protein kinase | 10.18 | 354.1 |
| A8K5H6 | | cDNA FLJ76659, highly similar to Homo sapiens exonuclease 1 (EXO1), transcript variant 2, mRNA | 10.14 | 93.8 |
| H0YKJ2 | | Solute carrier family 12 member 6 | 10.12 | 14.8 |
| A0A024QZH6 | | Serine arginine-rich pre-mRNA splicing factor SR-A1, isoform CRA_a | 10.11 | 139.2 |
| Q5T088 | | MORN repeat-containing protein 1 | 10.02 | 19.7 |
| A0A1W2PR28 | | IQ motif and SEC7 domain-containing protein 2 | 10.01 | 127.9 |
| Q9H6K5 | | Proline-rich protein 36 | 9.98 | 132.7 |
| Q5JW04 | | Solute carrier family 35 member C2 | 9.98 | 21.8 |
| O95425 | | Supervillin | 9.97 | 247.6 |
| Q5JPC9 | | ABI gene family, member 3 (NESH) binding protein, isoform CRA_d | 9.96 | 110.6 |
| A0A1W2PRA7 | | Calcium-activated potassium channel subunit alpha-1 | 9.96 | 107.9 |
| P16188 | | HLA class I histocompatibility antigen, A-30 alpha chain | 9.96 | 40.9 |
| P51826 | | AF4/FMR2 family member 3 | 9.93 | 133.4 |
| A0A087WU78 | | Nance-Horan syndrome protein | 9.93 | 157.7 |
| B4E063 | | Kinesin-like protein | 9.93 | 73.0 |
| A0A024RDD6 | | Uncharacterized protein | 9.87 | 82.4 |
| Q5VWG9 | | Transcription initiation factor TFIID subunit 3 | 9.84 | 103.5 |
| Q96JG9 | | Zinc finger protein 469 | 9.81 | 409.9 |
| Q14690 | | Protein RRP5 homolog | 9.78 | 208.6 |
| Q8NFC6 | | Biorientation of chromosomes in cell division protein 1-like 1 | 9.78 | 330.3 |
| O15457 | | MutS protein homolog 4 | 9.76 | 104.7 |
| A0A024RB02 | | PTPRF interacting protein, binding protein 1 (Liprin beta 1), isoform CRA_a | 9.75 | 96.9 |
| B4DYH4 | | cDNA FLJ51571, moderately similar to Mediator of DNA damage checkpoint protein 1 | 9.75 | 178.6 |
| Q5T7P2 | | Late cornified envelope protein 1A | 9.71 | 11.0 |
| A0A024R1T4 | | Trinucleotide repeat containing 6B, isoform CRA_b | 9.71 | 162.1 |
| Q9C0D5 | | Protein TANC1 | 9.68 | 202.1 |
| A0A0A0MTR7 | | E3 ubiquitin-protein ligase RNF213 | 9.67 | 591.0 |
| H0YLX2 | | DNA-binding protein RFX7 | 9.65 | 137.4 |
| S4R418 | | Bridging integrator 2 | 9.63 | 59.0 |
| Q9P206 | | Uncharacterized protein KIAA1522 | 9.61 | 107.0 |
| Q14395 | | Mucin | 9.59 | 51.9 |
| B2RWP0 | | Signal-induced proliferation-associated 1 like 3 | 9.58 | 194.5 |
| A2A2V2 | | RNA-binding protein 34 | 9.57 | 45.9 |
| A8K119 | | cDNA FLJ76742, highly similar to Homo sapiens deleted in liver cancer 1 (DLC1), transcript variant 2, mRNA | 9.55 | 122.7 |
| X6R7H2 | | Peroxisome proliferator-activated receptor gamma coactivator-related protein 1 | 9.53 | 69.1 |
| Q86UU5 | | Gametogenetin | 9.53 | 66.7 |
| Q8WY24 | | SNC66 protein | 9.48 | 53.6 |
| I0B0K6 | | Truncated profilaggrin | 9.46 | 277.1 |
| Q8N122 | | Regulatory-associated protein of mTOR | 9.44 | 148.9 |
| Q8NEY1 | | Neuron navigator 1 | 9.43 | 202.3 |
| Q86YZ3 | | Hornerin | 9.42 | 282.2 |
| Q9C0B5 | | Palmitoyltransferase ZDHHC5 | 9.41 | 77.5 |
| Q64FY1 | | AKNA transcript B1 | 9.38 | 146.2 |
| E7EPM4 | | Mucin-17 | 9.38 | 425.3 |
| B7ZKL5 | | AXIN2 protein | 9.34 | 86.6 |
| Q59FR9 | | Fibroblast growth factor 11 variant | 9.23 | 36.4 |
| A0A0A0MSP7 | | FERM and PDZ domain-containing protein 3 | 9.18 | 193.4 |
| A0A024RDF7 | | Uncharacterized protein | 9.17 | 130.2 |
| Q8IZF6 | | Adhesion G-protein coupled receptor G4 | 9.17 | 333.2 |
| H0Y785 | | Ankyrin repeat and KH domain-containing protein 1 | 9.14 | 107.8 |
| B8XCX8 | | EPC1/ASXL2b fusion protein | 9.12 | 201.7 |
| A0A087WX12 | | Centrosomal protein kizuna | 9.08 | 49.3 |
| Q8IZT6 | | Abnormal spindle-like microcephaly-associated protein | 9.07 | 409.5 |
| Q5KU26 | | Collectin-12 | 9.06 | 81.5 |
| P17948 | | Vascular endothelial growth factor receptor 1 | 8.97 | 150.7 |
| B3KVI8 | | cDNA FLJ16604 fis, clone TESTI4008097 | 8.95 | 157.9 |
| Q9Y6R7 | | IgGFc-binding protein | 8.87 | 571.6 |
| A0A140VJJ5 | | Testicular tissue protein Li 69 | 8.79 | 119.7 |
| A8K8Q0 | | cDNA FLJ78753, highly similar to Homo sapiens zinc fingers and homeoboxes 3 (ZHX3), mRNA | 8.76 | 104.6 |
| L8E9Z3 | | Alternative protein HRC | 8.45 | 34.3 |
| A0A126LB25 | | Immediate early protein IE2 | 8.44 | 164.2 |
| Q8IY33 | | MICAL-like protein 2 | 8.39 | 97.4 |
| B3KR06 | | CLIP-associating protein 2 | 8.38 | 49.1 |
| C9JG08 | | Uncharacterized protein C2orf16 | 8.19 | 598.1 |
| Q9ULH0 | | Kinase D-interacting substrate of 220 kDa | 8.15 | 196.4 |
| E1A689 | | Mutant Apo B 100 | 8.09 | 489.5 |
| Q12955 | | Ankyrin-3 | 7.98 | 480.1 |
| Q53QN0 | | Putative uncharacterized protein GTF3C2 | 7.78 | 58.4 |
| Q96BY7 | | Autophagy-related protein 2 homolog B | 7.75 | 232.6 |
| B0QYZ7 | | Eukaryotic translation initiation factor 4E transporter | 7.56 | 26.0 |
| Q20BI6 | | Cystic fibrosis transmembrane conductance regulator | 7.50 | 150.1 |
| Q96MW7 | | Tigger transposable element-derived protein 1 | 7.38 | 67.3 |
| B3KWK5 | | cDNA FLJ43230 fis, clone HCHON2001269 | 7.36 | 24.2 |
| E3W980 | | Helicase POLQ-like | 7.34 | 116.7 |
| Q45KX0 | | Brevideltin | 7.33 | 22.1 |
| Q8N2C7 | | Protein unc-80 homolog | 7.28 | 363.2 |
| B4DYX2 | | cDNA FLJ51404, highly similar to Netrin receptor DCC | 7.27 | 99.0 |
| F2Z2B6 | | Protein Jade-3 | 7.24 | 13.1 |
| B4E2K8 | | cDNA FLJ61075, highly similar to Mineralocorticoid receptor | 7.17 | 67.8 |
| H0YMD1 | | Low-density lipoprotein receptor | 7.14 | 104.7 |
| O75132 | | Zinc finger BED domain-containing protein 4 | 7.12 | 130.2 |
| H0YM61 | | Transient receptor potential cation channel subfamily M member 1 | 7.12 | 57.3 |
| B4DWC0 | | cDNA FLJ58290, highly similar to Zinc finger MYM-type protein 6 | 7.09 | 96.9 |
| B4DNH7 | | cDNA FLJ60079, highly similar to Tetratricopeptide repeat protein 3 | 7.09 | 99.7 |
| E9PPY5 | | Mas-related G-protein-coupled receptor member X3 | 7.08 | 27.8 |
| K7EKF7 | | Voltage-dependent P/Q-type calcium channel subunit alpha-1A | 7.06 | 98.8 |
| A0A0U1RQK2 | | Casein kinase I isoform gamma-1 | 7.03 | 15.4 |
| H0UI11 | | Dopey family member 1, isoform CRA_a | 7.02 | 255.6 |
| A2NH55 | | Immunogobulin kappa, VJ region | 7.00 | 11.9 |
| Q8N7X0 | | Androglobin | 6.99 | 189.6 |
| Q6ZS54 | | cDNA FLJ45821 fis, clone NT2RP8001584 | 6.96 | 79.2 |
| Q5T4T1 | | Transmembrane protein 170B | 6.96 | 14.4 |
| B4E0Q3 | | cDNA FLJ51432, highly similar to Dynamin-binding protein | 6.96 | 57.9 |
| A0A096LNL9 | | Transcriptional regulator ATRX | 6.94 | 151.6 |
| Q8N9U0 | | Tandem C2 domains nuclear protein | 6.92 | 55.2 |
| A0A087WW06 | | Tetratricopeptide repeat protein 28 | 6.92 | 256.7 |
| F2Z357 | | Rap1 GTPase-activating protein 1 | 6.91 | 66.9 |
| B4DMP4 | | cDNA FLJ53136, highly similar to Homo sapiens Vac14 homolog (VAC14), mRNA | 6.91 | 80.1 |
| A0A024R952 | | Plakophilin 1 (Ectodermal dysplasia/skin fragility syndrome), isoform CRA_a | 6.91 | 80.4 |
| B4DRA2 | | cDNA FLJ57828, highly similar to Treacle protein | 6.89 | 93.7 |
| A8K8T9 | | cDNA FLJ77187, highly similar to Homo sapiens cyclin B3 (CCNB3), transcript variant 3, mRNA | 6.88 | 157.8 |
| B3KX64 | | cDNA FLJ44873 fis, clone BRAMY2023939 | 6.87 | 77.9 |
| D6RFH5 | | Folliculin-interacting protein 2 | 6.86 | 74.7 |
| B3KV77 | | cDNA FLJ16222 fis, clone CTONG3002947 | 6.85 | 56.3 |
| D6RBK6 | | Type-1 angiotensin II receptor-associated protein | 6.85 | 16.4 |
| A0A1B0GUW1 | | Phosphatidylinositol N-acetylglucosaminyltransferase subunit Q | 6.85 | 6.9 |
| B3KXH9 | | cDNA FLJ45423 fis, clone BRHIP3036936 | 6.84 | 110.1 |
| H0YHI8 | | Protein phosphatase 1 regulatory subunit 12A | 6.83 | 40.6 |
| B4DG67 | | cDNA FLJ58842, highly similar to Homo sapiens zinc and ring finger 1 (ZNRF1), mRNA | 6.83 | 21.8 |
| Q5SWA1 | | Protein phosphatase 1 regulatory subunit 15B | 6.82 | 79.1 |
| B4DLG2 | | cDNA FLJ58196, highly similar to Zinc finger CCCH domain-containing protein 11A | 6.81 | 82.7 |
| Q6ZVL6 | | UPF0606 protein KIAA1549L | 6.81 | 198.9 |
| Q6WRX3 | | Protein zyg-11 homolog A | 6.79 | 85.8 |
| Q59FL0 | | Misshapen/NIK-related kinase isoform 2 variant | 6.78 | 93.2 |
| D3DTH7 | | Myosin IC, isoform CRA_a | 6.77 | 98.9 |
| Q9HBR1 | | Putative uncharacterized protein | 6.77 | 50.4 |
| Q71F56 | | Mediator of RNA polymerase II transcription subunit 13-like | 6.77 | 242.4 |
| C9J0I9 | | Nuclear-interacting partner of ALK | 6.76 | 50.5 |
| Q8N237 | | cDNA FLJ34965 fis, clone NTONG2004308 | 6.76 | 99.0 |
| A0A024RD26 | | G protein-coupled receptor 116, isoform CRA_a | 6.75 | 149.3 |
| S4R3C2 | | Nucleolar and coiled-body phosphoprotein 1 | 6.75 | 29.3 |
| A0A1W2PPB5 | | Bone morphogenetic protein receptor type-2 | 6.74 | 57.0 |
| C9JJU3 | | Bromodomain testis-specific protein | 6.74 | 52.8 |
| A0A0C4DFX2 | | Protein furry homolog | 6.74 | 338.0 |
| Q7Z408 | | CUB and sushi domain-containing protein 2 | 6.73 | 379.8 |
| G3V1X1 | | Proline/serine-rich coiled-coil 2, isoform CRA_a | 6.72 | 39.1 |
| Q68D69 | | Putative uncharacterized protein DKFZp779G1236 | 6.72 | 113.3 |
| Q9BU23 | | Lipase maturation factor 2 | 6.71 | 79.6 |
| B2RMV2 | | CYTSA protein | 6.71 | 124.5 |
| P08217 | | Chymotrypsin-like elastase family member 2A | 6.71 | 28.9 |
| Q5VV42 | | Threonylcarbamoyladenosine tRNA methylthiotransferase | 6.69 | 65.1 |
| E7EX48 | | Serine/threonine-protein kinase Nek4 | 6.69 | 80.6 |
| E9PL24 | | Myomegalin | 6.69 | 126.9 |
| Q9C0D2 | | Centrosomal protein of 295 kDa | 6.68 | 295.0 |
| A8KAE4 | | cDNA FLJ75520 | 6.68 | 131.2 |
| C9JN15 | | Peptidyl-prolyl cis-trans isomerase | 6.67 | 27.4 |
| C9JG84 | | Sorbin and SH3 domain-containing protein 2 | 6.67 | 12.3 |
| Q5JPB2 | | Zinc finger protein 831 | 6.66 | 177.8 |
| Q6NUQ2 | | Calmin (Calponin-like, transmembrane) | 6.66 | 111.6 |
| Q9ULL8 | | Protein Shroom4 | 6.65 | 164.8 |
| H0YBF7 | | Arf-GAP with SH3 domain, ANK repeat and PH domain-containing protein 1 | 6.65 | 105.2 |
| H9KVB3 | | Otogelin | 6.64 | 313.2 |
| A2RQD7 | | Bcr-abl1 e19a2 chimeric protein | 6.64 | 56.6 |
| P55291 | | Cadherin-15 | 6.64 | 88.9 |
| Q53H75 | | Chromosome 14 open reading frame 133 variant | 6.63 | 57.0 |
| B4DL51 | | cDNA FLJ60657, highly similar to Homo sapiens Smith-Magenis syndrome chromosome region, candidate 8 (SMCR8), mRNA | 6.63 | 75.3 |
| H3BU24 | | S phase cyclin A-associated protein in the endoplasmic reticulum | 6.63 | 6.4 |
| E9PML0 | | Cytochrome P450 4B1 | 6.62 | 36.4 |
| A5PLN7 | | Protein FAM149A | 6.61 | 82.6 |
| Q59G99 | | Dishevelled 1 isoform a variant | 6.61 | 40.3 |
| A0A140T8X5 | | STK19 | 6.61 | 40.4 |
| B2R621 | | cDNA, FLJ92736, highly similar to Homo sapiens gamma-aminobutyric acid (GABA) A receptor, alpha 3 (GABRA3), mRNA | 6.60 | 55.1 |
| A6NJB7 | | Proline-rich protein 19 | 6.60 | 38.7 |
| L8E8M9 | | Alternative protein PCDHB16 | 6.60 | 9.1 |
| B3KU03 | | cDNA FLJ39022 fis, clone NT2RP7003724 | 6.60 | 64.6 |
| Q9HCK8 | | Chromodomain-helicase-DNA-binding protein 8 | 6.59 | 290.3 |
| Q8IYW4 | | ENTH domain-containing protein 1 | 6.57 | 67.5 |
| Q2VIN3 | | RBM1 | 6.57 | 41.4 |
| B7ZKN6 | | UTX protein | 6.54 | 149.2 |
| F8WAI8 | | Zinc finger and BTB domain-containing protein 40 | 6.53 | 125.4 |
| Q13489 | | Baculoviral IAP repeat-containing protein 3 | 6.52 | 68.3 |
| H0Y482 | | Band 4.1-like protein 1 | 6.52 | 13.0 |
| Q53FJ3 | | Ubiquitin specific protease, proto-oncogene isoform a variant | 6.52 | 108.5 |
| Q7Z540 | | Mucin short variant SV7 | 6.50 | 13.4 |
| E9PNK1 | | Sialidase-3 | 6.50 | 18.8 |
| H0YGG9 | | Solute carrier organic anion transporter family member | 6.49 | 52.7 |
| Q5M9N0 | | Coiled-coil domain-containing protein 158 | 6.48 | 127.1 |
| D6RFZ4 | | Protein FAM193A | 6.48 | 87.2 |
| Q9P2Q4 | | 5-hydroxytryptamine (Serotonin) receptor 1F | 6.48 | 41.6 |
| Q68DX6 | | Putative uncharacterized protein DKFZp686P0776 | 6.46 | 78.0 |
| B0QZ65 | | GTPase-activating protein and VPS9 domain-containing protein 1 | 6.45 | 56.4 |
| B4DX00 | | cDNA FLJ61440, highly similar to Izumo sperm-egg fusion protein 1 | 6.45 | 26.4 |
| Q9Y6R1 | | Electrogenic sodium bicarbonate cotransporter 1 | 6.45 | 121.4 |
| Q96GX5 | | Serine/threonine-protein kinase greatwall | 6.44 | 97.3 |
| B4E124 | | Ankyrin repeat and LEM domain-containing protein 1 | 6.43 | 46.6 |
| B4DR76 | | cDNA FLJ58249, highly similar to Eukaryotic translation initiation factor 4E transporter | 6.43 | 68.9 |
| A8K482 | | Aspartate aminotransferase | 6.43 | 47.5 |
| Q8IY92 | | Structure-specific endonuclease subunit SLX4 | 6.43 | 199.9 |
| Q7Z2Z1 | | Treslin | 6.43 | 210.7 |
| Q8N397 | | Putative uncharacterized protein DKFZp761M142 | 6.42 | 89.8 |
| Q9H195 | | Mucin-3B | 6.42 | 131.3 |
| B3KUT9 | | cDNA FLJ40599 fis, clone THYMU2011183, highly similar to Thymus-specific serine protease (EC3.4.-.-) | 6.41 | 27.5 |
| A7MD48 | | Serine/arginine repetitive matrix protein 4 | 6.41 | 68.5 |
| A0A1B0GTR8 | | Ankyrin repeat and fibronectin type-III domain-containing protein 1 | 6.40 | 56.4 |
| B7Z5R7 | | cDNA FLJ61355, highly similar to CLIP-associating protein 1 | 6.40 | 135.7 |
| Q8IX28 | | SE2-5LT1 protein | 6.39 | 89.6 |
| O43147 | | Small G protein signaling modulator 2 | 6.39 | 113.2 |
| B4DNH6 | | Perilipin | 6.39 | 38.4 |
| F8VY01 | | FYVE, RhoGEF and PH domain-containing protein 6 | 6.39 | 135.4 |
| B2RU27 | | Testis expressed 15 | 6.38 | 315.1 |
| Q9ULK2 | | Ataxin-7-like protein 1 | 6.38 | 91.5 |
| A0A1W2PQT4 | | KAT8 regulatory NSL complex subunit 1 | 6.38 | 58.4 |
| A6ND36 | | Protein FAM83G | 6.36 | 90.8 |
| Q8N4A7 | | Putative uncharacterized protein | 6.36 | 8.0 |
| Q9BX26 | | Synaptonemal complex protein 2 | 6.35 | 175.5 |
| H0YCA1 | | Plasma protease C1 inhibitor | 6.34 | 9.9 |
| B3KS23 | | cDNA FLJ35336 fis, clone PROST2015464 | 6.34 | 56.4 |
| A0A1S5UZH2 | | Protein-methionine sulfoxide oxidase | 6.34 | 116.8 |
| G0Z071 | | Sex-determining region Y protein | 6.33 | 23.8 |
| L8E9J7 | | Alternative protein GPR78 | 6.33 | 9.6 |
| B5MCY1 | | Tudor domain-containing protein 15 | 6.32 | 221.6 |
| H0YAA7 | | Endomucin | 6.32 | 8.0 |
| Q8WYX5 | | Putative uncharacterized protein pp10472 | 6.31 | 17.9 |
| I3L209 | | Transforming growth factor beta-1-induced transcript 1 protein | 6.31 | 6.5 |
| Q53TA0 | | Receptor protein-tyrosine kinase | 6.30 | 79.0 |
| H7BXS9 | | Trinucleotide repeat-containing gene 18 protein | 6.29 | 42.6 |
| H0Y5R1 | | Aryl hydrocarbon receptor nuclear translocator-like protein 2 | 6.29 | 65.6 |
| Q9BRL6 | | Serine/arginine-rich splicing factor 8 | 6.23 | 32.3 |
| Q96KW2 | | POM121-like protein 2 | 6.23 | 109.8 |
| H0Y7V4 | | Dynein heavy chain 8, axonemal | 6.23 | 478.6 |
| A0A075B7B7 | | GAS2-like protein 2 | 6.19 | 94.8 |
| B6RC65 | | Epidermal growth factor receptor variant EX12_14del | 6.18 | 15.0 |
| B4DDG9 | | cDNA FLJ53856, highly similar to 5-aminolevulinate synthase, nonspecific, mitochondrial (EC 2.3.1.37) | 6.17 | 48.8 |
| Q684P5 | | Rap1 GTPase-activating protein 2 | 6.16 | 80.0 |
| Q9UBL0 | | cAMP-regulated phosphoprotein 21 | 6.15 | 89.1 |
| Q9BW04 | | Specifically androgen-regulated gene protein | 6.15 | 63.9 |
| A0A0G2JN42 | | Mucin-6 | 6.14 | 256.9 |
| Q2XPN4 | | Mitochondrial A kinase PPKA anchor protein 10 | 6.08 | 67.7 |
| Q9UND0 | | Killer inhibitory receptor 2-2-1 | 6.06 | 14.3 |
| E7EU81 | | Golgin subfamily B member 1 | 6.05 | 188.1 |
| H0UIC5 | | Ecotropic viral integration site 2B, isoform CRA_a | 6.04 | 39.9 |
| B4E1T1 | | cDNA FLJ54081, highly similar to Keratin, type II cytoskeletal 5 | 6.04 | 58.8 |
| P46013 | | Proliferation marker protein Ki-67 | 6.04 | 358.5 |
| O43419 | | Intestinal mucin | 6.02 | 60.5 |
| Q9NWN3 | | F-box only protein 34 | 6.02 | 78.7 |
| A0A024R8E8 | | Senataxin, isoform CRA_b | 6.01 | 199.9 |
| B7Z5J1 | | cDNA FLJ59265, highly similar to NAD-dependent deacetylase sirtuin-3, mitochondrial (EC 3.5.1.-) | 6.01 | 37.6 |
| B3KME0 | | cDNA FLJ10760 fis, clone NT2RP3004618 | 6.01 | 88.7 |
| Q59FF8 | | CUB and Sushi multiple domains 1 variant | 6.00 | 323.3 |
| A0A024R3H2 | | Sortilin-related receptor, L(DLR class) A repeats-containing, isoform CRA_b | 5.98 | 248.3 |
| E9PHY1 | | Low-density lipoprotein receptor-related protein 5 | 5.96 | 52.4 |
| C9JT44 | | Solute carrier family 25 member 38 | 5.95 | 16.6 |
| B3KR17 | | cDNA FLJ33465 fis, clone BRAMY2001367 | 5.94 | 86.5 |
| Q6ZR29 | | cDNA FLJ46702 fis, clone TRACH3014183 | 5.91 | 160.1 |
| D6RDI1 | | E3 ubiquitin-protein ligase MIB2 | 5.90 | 11.6 |
| Q05DV5 | | ZC3HAV1 protein | 5.90 | 49.2 |
| B4DS83 | | cDNA FLJ53179, highly similar to Nucleolar protein 10 | 5.88 | 74.4 |
| D2CPJ9 | | Mutant mutant xeroderma pigmentosum complementation group C protein | 5.84 | 25.7 |
| Q6ZU64 | | Cilia- and flagella-associated protein 65 | 5.83 | 217.1 |
| B2R8T0 | | cDNA, FLJ94049, highly similar to Homo sapiens egf-like module containing, mucin-like, hormonereceptor-like sequence 1 (EMR1), mRNA | 5.82 | 97.7 |
| A8K857 | | cDNA FLJ76361 | 5.82 | 23.5 |
| B7Z7H2 | | cDNA FLJ58079, highly similar to Homo sapiens SH3 multiple domains 1 (SH3MD1), mRNA | 5.81 | 106.3 |
| P0C7P2 | | Putative protein RFPL3S | 5.81 | 11.7 |
| H0YLM8 | | DmX-like protein 2 | 5.80 | 132.6 |
| B4DG65 | | cDNA FLJ52105, highly similar to PDZ domain-containing RING finger protein 4 | 5.79 | 89.0 |
| K4DI93 | | Cullin 4B, isoform CRA_e | 5.71 | 102.7 |
| A8K601 | | cDNA FLJ75186 | 5.70 | 118.4 |
| B7Z3E3 | | Reticulon | 5.69 | 100.8 |
| P27815 | | cAMP-specific 3',5'-cyclic phosphodiesterase 4A | 5.69 | 98.1 |
| A0A1B0GTP3 | | Long intergenic non-protein coding RNA 238 | 5.64 | 18.9 |
| Q86U11 | | Full-length cDNA clone CS0DE006YM09 of Placenta of Homo sapiens (human) | 5.47 | 47.7 |
| A0A0B4J2E9 | | High affinity immunoglobulin epsilon receptor subunit beta | 5.30 | 21.6 |
| K7EIU2 | | Serine/threonine-protein kinase ULK2 | 5.26 | 21.0 |
| B2R7I0 | | cDNA, FLJ93451, highly similar to Homo sapiens thioredoxin domain containing 14 (TXNDC14), mRNA | 5.23 | 34.0 |
| Q5VSN0 | | SH3 domain-containing kinase-binding protein 1 | 5.22 | 61.4 |
| A0A087WVA8 | | Testis-expressed protein 2 | 5.22 | 125.3 |
| F8WF20 | | Integrin beta-1-binding protein 1 | 5.21 | 4.3 |
| F5GYN0 | | Protein FAM186A | 5.09 | 262.1 |
| B3KN94 | | cDNA FLJ13982 fis, clone Y79AA1001711 | 5.05 | 62.0 |
| E9PBD8 | | Lymphocyte-specific protein 1 | 4.85 | 24.4 |
| Q59EZ3 | | Insulin-like growth factor 2 receptor variant | 4.69 | 265.9 |
